# Patient-relevant outcomes: what are we talking about? A scoping review to improve conceptual clarity

**DOI:** 10.1186/s12913-020-05442-9

**Published:** 2020-06-29

**Authors:** Christine Kersting, Malte Kneer, Anne Barzel

**Affiliations:** 1 Institute of General Practice and Interprofessional Care, Faculty of Health/School of Medicine, Witten/Herdecke University, Alfred-Herrhausen-Str. 50, 58448 Witten, Germany; 2 Institute of General Medicine, Ulm University, Albert-Einstein-Allee 23, 89081 Ulm, Germany

**Keywords:** Patient-relevant outcome, Patient relevance, Patient preference, Patient-centered care, Patient involvement

## Abstract

**Background:**

With respect to patient-centered care, measuring care effects based on patient-relevant outcomes is becoming increasingly important. There is some uncertainty about what outcomes are particularly relevant to patients and who determines their relevance. To determine this, we conducted a scoping review of the international literature with the aim to improve the conceptual clarity regarding (1) the terminology used for supposedly patient-relevant outcomes, (2) the variety of outcomes considered patient-relevant, and (3) justifications for the choice of these specific outcomes.

**Methods:**

We conducted a systematic search in Embase, PubMed (including Medline), Cochrane Central, Scopus, and Google Scholar with a special focus on article titles. Search terms included patient-relevant, patient-important, patient-preferred, and outcome(s), endpoint(s), parameter(s), indicator(s). We limited the search period from January 2000 to July 2019. Full-text articles reporting outcomes that were described as patient-relevant met the inclusion criteria. Two researchers independently analyzed all eligible articles applying quantitative and structuring content analysis.

**Results:**

We identified 155 articles, 44 of which met the inclusion criteria. A content analysis revealed 35 different terms used with regard to patient-relevant outcomes. However, authors predominantly referred to patient-important outcomes (23 articles, 52.3%) and patient-relevant outcomes (17 articles, 38.6%). A structuring content analysis of all extracted outcomes revealed a total of 281 codes, pooled in 32 inductive categories. Among these, the following categories dominated: symptoms, adverse events/complications, survival/mortality, pain. In just 16 of the articles (36.4%), authors provided justifications for the choice of the outcome being based either on patient and/or expert opinions. In another 13 articles (29.5%), no justification was provided.

**Conclusion:**

This scoping review on patient-relevant outcomes was driven by the questions (1) what outcomes are particularly relevant to patients, and (2) who determines their relevance. We found a wide range of supposedly patient-relevant outcomes, with only one third of articles involving patients in the justification of the outcome selection. In view of this conceptual uncertainty it appears difficult to determine or even to compare a particular patient benefit of interventions. A set of generic outcomes relevant to patients would be helpful to contribute to a consistent understanding of patient relevance.

## Background

Patient-centered care aims to place individuals, their values, preferences, life and health goals at the heart of the care process and to actively involve patients in care decisions [1]. In the sense of shared decision-making, involving patients in decisions implies that patients are adequately informed about existing care options and their potential effects, understand these options, and are given the opportunity to explore what is most relevant to them in order to make a choice based on their personal preferences [2]. In our understanding, this means that for shared decision-making, effect measurements based on parameters that matter to patients are urgently needed. This assumes that studies examine the effects of care that is based on outcomes which are relevant to patients, but so far systematic reviews conclude that such patient-relevant outcomes are underrepresented in recent clinical trials [3–5]. Indeed, this raises the question as to what outcomes are relevant to patients and who determines their relevance.

In Germany, the Institute for Quality and Efficiency in Health Care (IQEHC) officially examines the benefits and harms of medical interventions for patients. To this end, the IQEHC considers parameters to be relevant to patients when they represent how a patient feels, functions or survives; notably mortality, morbidity, and quality of life [6]. However, this understanding of patient relevance is based on a definition of the Biomarkers Definition Working Group on clinical endpoints, which does not contain any information about patient relevance [7]. Similarly to the IQEHC’s understanding of patient-relevant outcomes, the working group on quality of care and patient safety research of the German Network for Health Services Research (DNVF) mentions survival and quality of life as factors that are relevant to patients, but also includes social aspects, such as social reintegration, in its interpretation [8]. Generally, these outcomes are based on the understanding that patient-relevant outcomes reflect the effects of changes in the individual patient’s health status [8].

However, neither the definition provided by the IQEHC nor that provided by the working group of the DNVF offered us a comprehensive explanation as to why the outcomes mentioned are considered to be relevant to patients. Shifting the view from the German to the international context, we aimed to examine which understanding of patient relevance and which outcomes are common to international research and thereby hoped to improve the conceptual clarity of patient relevance. To this end, we conducted a scoping review of the international literature with regard to the following research questions: (1) What terminology is used for supposedly patient-relevant outcomes? (2) What outcomes are considered to be relevant to patients? (3) What explanations are provided to justify the relevance of these specific outcomes for patients?

## Methods

We report our scoping review in accordance with the PRISMA extension for scoping reviews (PRISMA-ScR) [9].

### Data sources and search strategy

We conducted a systematic literature search in Embase, PubMed (including Medline), Cochrane Central, Scopus, and Google Scholar. Our search strategy considered German and English references published between January 1st, 2000 and July 31st, 2019. Restricting the search period to the past 20 years was considered reasonable to concentrate on more recent research. In order to identify references that clearly focus on the topic of interest, we additionally restricted the search to the article titles. Titles had to include at least one of the following terms: *(patient-relevant OR patient-important OR patient-preferred) AND (outcome(s) OR endpoint(s) OR parameter(s) OR indicator(s)).* The full search strategies for each database are outlined in Additional file 1.

### Study selection

After deleting all duplicates, two of the researchers (Christine Kersting (CK), Malte Kneer (MK)) reviewed all records retrieved from the database search: In the first step, we independently screened the records to limit the search results to English- and German-language articles with full-text availability published in journals. This was reasonable as we aimed to examine whether there is, in fact, a common and consistent understanding of patient relevance in international research. Accordingly, we excluded doctoral theses if not officially published, conference abstracts, commentaries on previously published articles, opinions, debates, or editorials, records on other topics, and those without full-text availability. In the second step, we independently reviewed the full texts of the remaining articles to check whether they met the inclusion criteria for our review. We defined articles as eligible when they reported outcomes that were described by the authors as relevant to patients. In case of disagreements we discussed these until a consensus was achieved.

### Data extraction and analysis

In line with the three research questions of this scoping review, we analyzed all eligible articles regarding (1) the terminology used, (2) the outcomes described as patient-relevant, and (3) the justifications for why these outcomes were considered to be of relevance to patients. For the analysis we used both quantitative content analysis and qualitative structuring content analysis [10].

#### Terminology

All terms that were used to describe patient-relevant outcomes were assessed and extracted in original spelling to collect synonyms; however, paraphrases such as “outcomes that matter to patients” were not considered synonyms. Finally, we applied frequency calculations to quantify the number of different terms used per article and the overall number of different terms identified.

#### Outcomes

In a second step all outcomes described as relevant to patients were extracted from the articles and structured for the underlying issues (thematically) using inductive categories. The code set was extended continuously as new categories emerged during the full-text analyses. We additionally examined whether specific outcome categories were used more often than others by calculating frequencies and percentages.

#### Justification

We analyzed whether the articles provided a justification as to why the outcomes described were considered to be relevant to patients. These justifications were categorized inductively. Based on this, we distributed the outcome categories that were identified in the previous step to the justifications provided.

In a subgroup analysis we focused on articles that actively involved patients or experts in the definition of patient-relevant outcomes. We examined whether the outcomes defined in these articles were valid for specific patient groups only and whether these outcomes were different from those described as relevant to patients in articles that did not involve patients or experts. Additionally, we stratified for studies involving patients, studies involving experts, and those involving both, and compared the outcome categories between these groups.

## Results

### Literature search

**Fig. 1:**
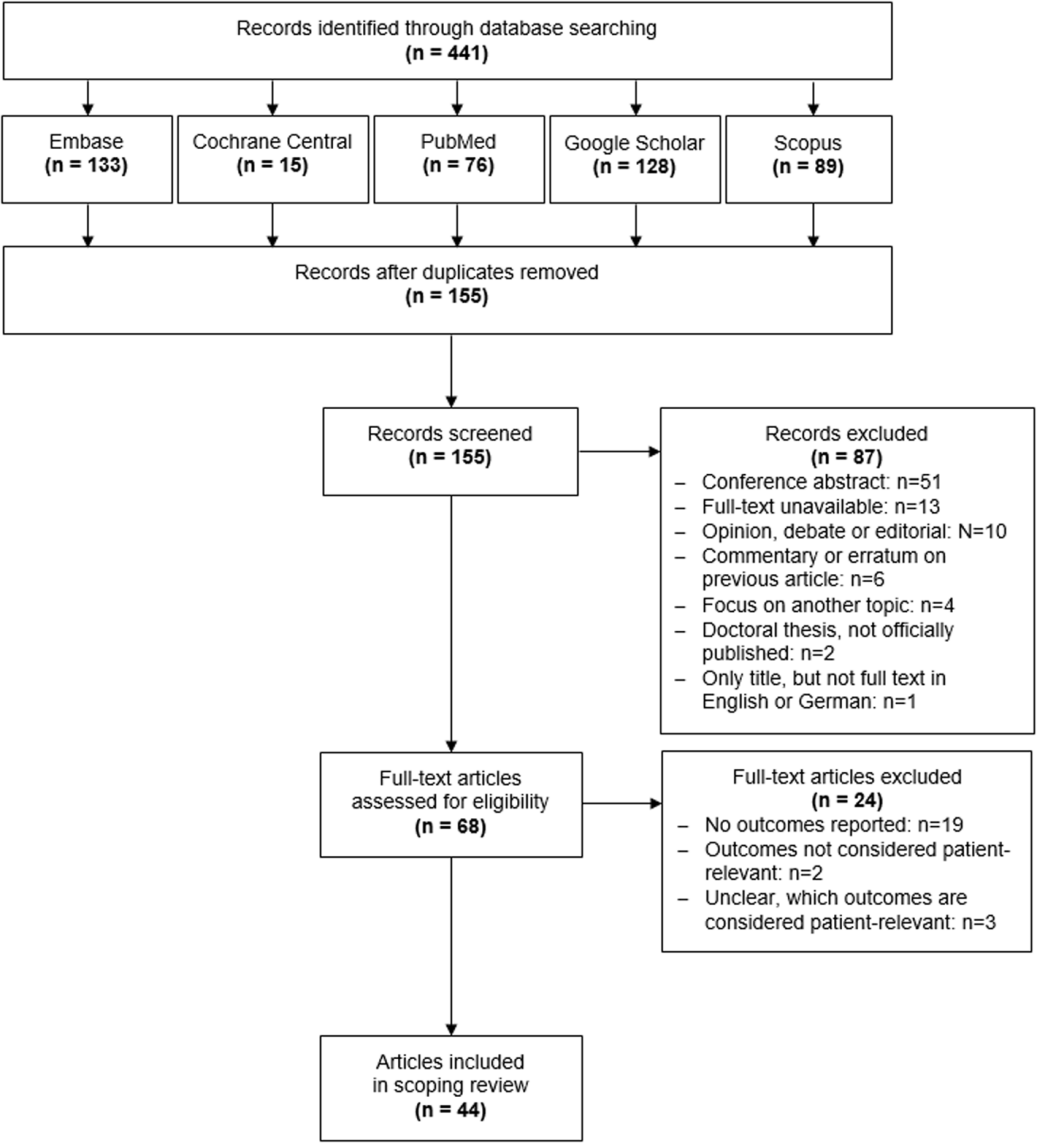
Flow chart depicting study selection

Excluding duplicates, the literature search yielded 155 records (Fig. 1). During the first screening, we excluded 87 records because the full-text papers were unavailable, not written in English or German, focused on another topic, or were conference abstracts, commentaries on previously published articles, opinions, debates, or editorials. Full-text screening of the remaining 68 articles resulted in 44 articles fulfilling the inclusion criteria for this review. Details on all studies are provided in Tables 1, 2, 3 and 4.

**Table 1:** Characteristics of the 16 articles involving patients and/or experts to justify patient relevance of outcomes

Authors	Objective/ motivation of article	Type of article/ study	Terms used synonymously	Indicator disease/intervention	Outcomes considered patient-relevant [instrument, if applicable]
Blome et al. 2009 [11]	Development and validation of a specific version of the German patient benefit index (PBI-S) to be used in the treatment of pruritus (PBI-P)	Mixed methods study involving patients and experts	5 terms: - patient-relevant benefit - patient-relevant treatment benefit - patient preference - patient-reported benefit - patient-relevant outcome	Pruritus	In decreasing order of importance, i.a.: - no longer experience itching - find a clear diagnosis and therapy - have confidence in therapy - be free of pain - no longer have a burning sensation on the skin - be able to sleep better - be less dependent on doctor and clinic visits
Blome et al. 2014 [12]	Development and validation of a specific version of the German patient benefit index (PBI-S) to be used in the treatment of lymphedema and lipedema (PBI-L)	Mixed methods study involving patients and experts	3 terms: - patient-relevant outcome - patient-relevant benefit - patient-relevant treatment benefit	Lymphedema, lipedema	In decreasing order of importance, i.a.: - experience less swelling and tension - be free of pain - avoid complications - receive optimal hosiery (e.g., color, fit, prescription quantity) - be less restricted in your ability to move around - have no fear that the disease will become worse - find a clear diagnosis and therapy
Cho et al. 2019 [13]	Identification of patients‘and caregivers‘priorities for outcomes in trials on autosomal dominant polycystic kidney disease	Mixed methods study involving patients only	4 terms: - patient-important outcome - patient-prioritised outcome - patient-centered outcome - patient-reported outcome	Autosomal dominant polycystic kidney disease	In decreasing order of importance, i.a.: - kidney function - end-stage kidney disease - survival - cyst size/growth - cyst pain/bleeding - blood pressure - ability to work - cerebral aneurysm/stroke - mobility/physical function - fatigue
Daeter et al. 2018 [14]	1. Share the standard set of outcomes for coronary artery disease developed by Meetbaar Beter 2. Illustrate how the standard set is presented and published Note: Meetbaar Beter aims to improve the quality of cardiovascular care in hospitals in the Netherlands by creating transparency on patient-relevant outcomes (Benchmarking)	Delphi approach among experts only	1 term: - patient-relevant outcome	Coronary artery disease (overall)	- long-term survival (≤5 years) - 1-year mortality - quality of life [SF-36] - myocardial infarction (≤30 days)
				Coronary artery bypass grafting	- 120-day mortality - surgical reexploration (≤30 days) - cerebrovascular accident (≤72 h) - Deep sternal wound infection (≤30 days) - free of myocardial infarction - free of coronary artery reintervention
				Percutaneous coronary intervention	- 30-day mortality - urgent coronary artery bypass grafting (≤24 h) - occurrence of target vessel revascularization (≤1 year)
				Conservative treatment	- chest pain (≤1 year) - free of major adverse cardiac event
Dinglas et al. 2018 [15]	Synthesis of the literature with regard to patient-important outcome for Intensive Care Unit survivors focusing on a research program on acute respiratory failure	Synthesis, Delphi approach among patients only	1 term: - patient-important outcome	Acute respiratory failure	- survival - physical function - muscle and/or nerve function - pulmonary function - cognition - mental health [HADS, IES-R] - pain [EQ-5D pain question] - health-related quality of life [EQ-5D, SF-36]
Eiring et al. 2016 [16]	1. Investigate the relative importance of patient-important outcomes in bipolar disorder 2. Construct a holistic taxonomy of patient-important outcomes	Mixed methods study involving patients only	2 terms: - patient-important outcome - patient-relevant outcome	Bipolar disorder	In decreasing order of importance, i.a.: - avoid serve depression - avoid serve mania - increase quality of life - better functioning (school/work) - better social functioning
Kinter et al. 2009 [17]	1. Identification of endpoints directly from patients with schizophrenia 2. Evaluation whether patients can express which endpoints matter to them 3. Ranking of the relevant endpoints	Mixed methods study involving patients only	5 terms: - patient-relevant endpoint - patient-relevant benefit - patient endpoint - patient preference - patient-centered endpoint	Schizophrenia	In decreasing order of importance, i.a.: - clear thinking - minimization of symptoms - social activities - daily activities - supportive physician
Konkle et al. 2019 [18]	Review of strengths and limitations of outcome measures used in hemophilia trials from a provider and patient perspective	Review involving patients and experts	3 terms: - patient-important outcome - patient-relevant outcome - patient-important clinical outcome	Hemophilia	- frequency of bleeds - factor activity level - duration of expression - chronic pain - healthcare resource use - mental health
Lindsley et al. 2018 [19]	Identification and prioritization of clinical questions and patient-relevant outcomes for research associated with the treatment of age-related macular degeneration	Delphi approach among patients and experts	1 term: - patient-important outcome	Age-related macular degeneration	Highly important to patients: - choroidal neovascularization - development of advanced age-related macular degeneration - retinal hemorrhage - gain of vision - slowing vision loss - serious ocular events
Murad et al. 2011 [20]	Assessment of preferences of patients with diabetes on how clinical trials should be conducted with regard to study design (pragmatic versus explanatory) and endpoints (surrogate versus patient-relevant)	Cross-sectional study involving patients only	2 terms: - patient-important outcome - patient preference	Diabetes mellitus	In decreasing order of importance, i.a.: - end-stage renal disease - stroke - myocardial infarction - blindness - HbA1c control - death
Nabbout et al. 2018 [21]	Identification of a core set of patient- and caregiver-relevant concepts to be included in future clinical trials on dravet syndrome	Qualitative study, Delphi approach among caregivers and experts	2 terms: - patient- and caregiver-relevant outcome - patient- and caregiver-relevant endpoint	Dravet syndrome	- seizures - expressive communication of the child - receptive communication of the child - daily activities of the caregiver - social functioning of the caregiver
Sanderson et al. 2010 [22]	Identification of treatment outcomes important to patients with rheumatoid arthritis receiving anti-tumor necrosis factor therapy	Qualitative study involving patients only	6 terms: - patient outcome - patient priority treatment outcome - patient priority outcome - patients’ important treatment outcome - patients’ important outcome - patient-important outcome	Rheumatoid arthritis	- rheumatoid arthritis under control (symptoms less, rheumatoid arthritis stable, medication effects) - doing things (doing things, able to plan) - emotional health (positive feelings, holistic identity, positive mental changes, better life) - coping with illness (coping with rheumatoid arthritis, coping with health system) - global outcomes (feeling well, return to a normal life, feeling (more) normal)
Sung et al. 2014 [23]	Development of a comprehensive conceptual framework representing the relevant dimensions and outcomes important to women with pelvic organ prolapse	Mixed methods study involving patients only	1 term: - patient-important outcome	Pelvic organ prolapse	In decreasing order of importance, i.a.: - alleviation of physical bulge symptoms and associated discomfort - improvement in physical function - improvement in sexual function - improvement in body image perception - improvement in social function
Van der Elst et al. 2016 [24]	Better comprehension of the perspective of patients with early rheumatoid arthritis on preferred health and treatment outcomes	Qualitative study involving patients only	2 terms: - patient-preferred outcome - patient-preferred health and treatment outcome	Early rheumatoid arthritis	- aspects of disease control, e.g., prevention or control of joint damage, less medication - physical aspects, e.g., relief of pain and other physical symptoms, improved joint function and mobility - aspects of participation, e.g., performing activities of daily living, engaging in work and/or leisure - mental aspects, e.g., emotional well-being, life enjoyment
Wilson et al. 2019 [25]	Synthesis of evidence in all outcome domains identified as important by patients undergoing unicompartmental or total knee replacement and outcome domains commonly used in other studies	Systematic review & meta-analysis involving patients and referring to other studies	1 term: - patient relevant outcome	Unicompartmental and total knee replacement in osteoarthritis	- hospital admission impact: length of operation, length of hospital stay - risk of early complications (myocardial infarction, stroke, venous thromboembolism, deep infection) or early mortality - success of operation: range of movement achieved or kneeling ability, reduction in pain, improvement in function - reoperation or revision rate - rate of recovery: rate of return to work, rate of return to sporting activities
Van Veghel et al. 2016 [26]	Presentation and discussion of the patient-relevant outcomes of Meetbaar Beter for coronary artery disease and aortic valve disease, focusing on the surgical procedures coronary artery bypass grafting, percutaneous coronary intervention, aortic valve replacement and transcatheter aortic valve implantation	Database analysis involving experts only	3 terms: - patient-relevant outcome - patient-relevant health outcome - patient-oriented outcome	Coronary artery disease (overall)	- readmission due to myocardial infarction (≤30 days)
				Coronary artery bypass grafting	- 120-day mortality - quality of life [SF-36]
				Percutaneous coronary intervention	- 1-year mortality - Occurrence of target vessel revascularization (≤1 year)
				Aortic valve replacement	- 120-day mortality - long-term survival
				Transcatheter aortic valve implantation	- 120-day mortality - implantation of a new permanent pacemaker (≤30 days)

**Table 2:** Characteristics of the 12 articles referring to other studies or special classifications to justify patient relevance of outcomes

Authors	Objective/ motivation of article	Type of article/ study	Terms used synonymously	Indicator disease/intervention	Outcomes considered patient-relevant [instrument, if applicable]
Adie et al. 2017 [4]	Determination of the proportion of patient-important primary outcomes in surgical randomized controlled trials	Systematic review & meta-analysis	2 terms: - patient-important outcome - patient-centered outcome	Surgical interventions	- mortality/survival - pain - function - quality of life - any morbid event or symptom - patient satisfaction - any intervention to address the previous outcomes
Ameur et al. 2017 [27]	Determination whether recently published an ongoing systematic reviews with meta-analyses of therapeutic interventions assess patient-important outcomes	Methodological review	1 term: - patient-important outcome	Generic; therapeutic interventions	- mortality - clinical events - pain - quality of life - therapeutic decision - function
Fei et al. 2018 [28]	Examination of the impact of adding ezetimibe to statins on patient-important outcomes in patients at high cardiovascular risk	Narrative systematic review	1 term: - patient-important outcome	High cardiovascular risk	- all-cause mortality - cardiovascular mortality - non-fatal stroke - non-fatal myocardial infarction - adverse events
Gaudry et al. 2017 [5]	Investigation whether randomized controlled trials in critically ill patients assess patient-important outcomes	Systematic review	1 term: - patient-important outcome	Critical illness	- mortality - quality of life after Intensive Care Unit discharge - functional, cognitive, and neurological outcomes after Intensive Care Unit discharge
Kvitkina et al. 2014 [29]	Description of the feasibility of the early benefit assessment of novel agents on the basis of patient-relevant outcomes by characterizing the outcomes available in the companies dossiers and comparing them to outcomes defined as patient-relevant by the German Institute for Quality and Efficiency in Health Care	Systematic Review	1 term: - patient-relevant outcome	Drugs (novel agents)	- mortality - morbidity - health-related quality of life - adverse events
Roos et al. 2000 [30]	Evaluation of patient-relevant outcomes preoperatively and three months after partial meniscectomy	Prospective follow-up study	1 term: - patient-relevant outcome	Partial meniscectomy	- general health status [SF-36] - knee-specific health status: pain, symptoms, activities of daily life, sports and recreation function, knee-related quality of life, functional disability [KOOS, Lysholm Knee Score]
Schumacher et al. 2016 [31]	Assessment of current approaches to measure the impact of tuberculosis nucleic acid amplification tests on patient-important outcomes in adults with possibly tuberculosis and/or drug-resistant tuberculosis	Methodological review	2 terms: - patient-important outcome - patient outcome	Tuberculosis nucleic acid amplification tests	- culture conversion - tuberculosis treatment outcomes - infection control/ contact tracing - morbidity - mortality
Singh et al. 2017 [32]	Simulation of the long-term effect of novel agents versus chemotherapy-based regimens on progression-free survival, overall survival and health-related quality of life in patients with chronic lymphocytic leukemia	Review & data simulation	2 terms: - patient-relevant outcome - patient-relevant benefit	Chronic lymphocytic leukemia	- progression-free survival - overall survival - quality-adjusted life years - post-progression survival
Wieseler et al. 2013 [33]	Determination of the information gain between clinical study reports and publicly available sources for patient-relevant outcomes included in health technology assessments for drugs	Systematic review	2 terms: - patient-relevant clinical trial outcome - patient-relevant outcome	Drugs	- mortality - clinical events - symptoms - health-related quality of life - (serious) adverse events
Yordanov et al. 2018 [34]	1. Evaluation whether the outcomes reported in the summary of finding table of Cochrane reviews could be considered patient-important 2. Evaluation of the quality of evidence for these outcomes	Methodological review	1 term - patient-important outcome / PIO	Generic	- mortality - other clinical events (e.g., myocardial infarction, stroke) - adverse events - function (e.g., anxiety, depression, disability) - pain - quality of life - therapeutic decisions
El Dib et al. 2017 [35]	Review of randomized controlled trials on diagnostic tests with regard to their topic areas, population, setting, study groups, patient-important outcomes, risk of bias, and results	Systematic review	1 term: - patient-important outcome	Diagnostic tests	- mortality - morbidity - symptoms - quality of life - functional status
Fayed et al. 2014 [36]	1. Review to which extent activity and participation outcomes are included in pediatric clinical trials 2. Determination what characteristics are associated with using theses outcomes	Systematic review	3 terms: - patient-important outcome - patient-important activity and participation outcome - child and family-important outcome	Generic; children with chronic conditions	- body function - activity - participation - environmental factors - personal factors - health condition - general health

**Table 3:** Characteristics of the 3 articles equating patient-relevant outcomes with self-reported outcomes

Authors	Objective/ motivation of article	Type of article/ study	Terms used synonymously	Indicator disease/ intervention	Outcomes considered patient-relevant [instrument, if applicable]
Nilsdotter et al. 2009 [37]	1. Description of outcomes up to five years after total knee replacement for osteoarthritis from the patients’ perspective 2. Evaluation to what extent patients performed physical activities after total knee replacement 3. Identification of preoperative characteristics predicting postoperative outcomes	Prospective follow-up study	2 terms: - patient-relevant outcome - self-reported outcome	Total knee replacement in osteoarthritis	- general health status [SF-36] - knee-specific health status: pain, stiffness, physical function [WOMAC] - general comorbidity
Nilsdotter, Isaksson 2010 [38]	Prospective evaluation of patient-relevant outcomes seven years after total hip replacement for osteoarthritis focusing on pain and physical function	Prospective cohort study with matched controls without hip complaints	2 terms: - patient-relevant outcome - patient-reported outcome	Hybrid total hip replacement and demented total hip replacement for osteoarthritis	- general health status [SF-36] - knee-specific health status: pain, stiffness, physical function [WOMAC] - postoperative complications - general comorbidity - musculoskeletal comorbidity: need of walking assistance, walking distance, pain, need for analgesics, joint replacement in contralateral hip or in knee, fractures - patient satisfaction
Nilsdotter, Lohmander 2003 [39]	Investigation of pre- and postoperative patient-relevant outcomes between hybrid total hip replacement and cemented total hip replacement in patients with osteoarthritis	Prospective cohort study	2 terms: - patient-relevant outcome - patient-relevant measure	Total hip replacement for osteoarthritis	- general health status [SF-36] - knee-specific health status: pain, stiffness, physical function [WOMAC] - postoperative complications - general comorbidity - musculoskeletal comorbidity: need of walking assistance, walking distance, pain, need for analgesics, joint replacement in contralateral hip or in knee, fractures

**Table 4:** Characteristics of the 13 studies not providing any justification for the patient relevance of outcomes

Authors	Objective/ motivation of article	Type of article/ study	Terms used synonymously	Indicator disease/ intervention	Outcomes considered patient-relevant [instrument, if applicable]
Agarwal et al. 2017 [40]	Examination to what extent Cochrane and non-Cochrane reviews report absolute effects for patient-important outcomes in the abstract	Systematic review	1 term: - patient-important outcome	Generic	- mortality - morbidity - symptoms - quality of life - functional status
Cao et al. 2014 [41]	Comparison of the efficacy of two commonly used Chinese patent medicines for patients with angina pectoris	Study protocol for a randomized controlled trial	1 term: - patient-important outcome	Angina pectoris	- short of breath - fatigue - palpitations - sweating
Cleveringa et al. 2010 [42]	Determination of the effects of the Diabetes Care Protocol on patient-important outcomes Note: When applying the Diabetes Care Protocol, routine diabetes care is delegated to a nurse, who uses a computerized decision support system to structure diabetes care and set targets	Cluster-randomized trial	1 term: - patient-important outcome	Type 2 diabetes	- diabetes-specific health status: psychological distress, barriers to activity, disinhibited eating [DHP-18] - general health status [SF-36, EQ-5D] - treatment satisfaction [DTSQ-status] - self-efficacy [DES-SF]
Englund et al. 2001 [43]	Evaluation of long-term patient-relevant outcomes after removal of knee meniscus	Retrospective cohort study	2 terms: - patient-relevant outcome - self-administered outcome measure	Meniscectomy	- general health status [SF-36] - knee-specific health status: pain, symptoms, activities of daily life, sports and recreation function, knee-related quality of life [KOOS]
Gandhi et al. 2008 [3]	Determination to what extent registered randomized controlled trials among patients with diabetes plan to assess patient-important outcomes	Systematic review	1 term: - patient-important outcome	Diabetes	- mortality - quality of life - major morbid events - minor morbid events - pain - functional status
Griffith et al. 2019 [44]	Determination how different disease frameworks impact the prevalence of multimorbidity and its association with patient-important outcomes	Baseline analysis of a population-based cohort study	2 terms: - patient-important functional outcome - patient-important outcome	Generic; community-living adults aged 45 to 85 years	- functional disability [OARS questionnaire] - social participation restriction - self-rated physical health - self-rated mental health
Nilsdotter et al. 2003 [45]	Evaluation of long-term patient-relevant outcomes after unilateral total hip replacement for osteoarthritis	Case-control-study	1 term: - patient relevant outcome	Unilateral total hip replacement for osteoarthritis	- general health status [SF-36] - knee-specific health status: pain, stiffness, physical function [WOMAC] - postoperative complications - general comorbidity - musculoskeletal comorbidity: need of walking assistance, walking distance, pain, need for analgesics, joint replacement in contralateral hip or in knee, fractures - patient satisfaction
Paradowski et al. 2004 [46]	Assessment of variation in knee pain, function, and quality of life over two years after removal of knee meniscus in patients with and and without radiographic knee osteoarthritis	Prospective follow-up study	1 term: - patient-relevant outcome	Meniscectomy	- knee-specific health status: pain, symptoms, activities of daily life, sports and recreation function, knee-related quality of life [KOOS]
Porat et al. 2004 [47]	Identification of the consequences of an anterior cruciate ligament tear 14 years after injury in a cohort of male soccer players regarding radiographic knee osteoarthritis and patient-relevant outcomes	Prospective cohort study	1 term: - patient relevant outcome	Anterior cruciate ligament tear	- general health status [SF-36] - knee-specific health status: pain, symptoms, activities of daily life, sports and recreation function, knee-related quality of life, functional disability [KOOS, Lysholm Knee Score]
Ramar et al. 2017 [48]	Synthesis of the literature with regard to models of care targeting patient-important outcomes for maintenance dialysis patients focusing on hospitalization and mortality	Systematic review & meta-analysis	2 terms: - patient-important outcome - patient outcome	Maintenance dialysis care	- mortality - hospitalization
Schnabel et al. 2014 [49]	Comparison of the analgesic efficacy and safety of ultrasound and nerve stimulator guided peripheral nerve catheters for postoperative pain therapy	Retrospective database analysis	4 terms: - patient-relevant target parameter - patient-related outcome - patient-relevant parameter *[German: Patienten-relevanter Parameter]* - patient-relevant efficacy parameter *[German: Patienten-relevanter Effektivitätsparameter]*	Peripheral nerve catheters for pain therapy	- postoperative pain - postoperative need for additional opioids - cumulative local anesthetic consumption - puncture-associated complications - postoperative catheter-related complications
Stallmach et al. 2015 [50]	Examination of possible improvements in the clinical situation of patients with inflammatory bowel disease in Germany, focusing on patient-relevant endpoints	Secondary data analysis	1 term: - patient-relevant endpoint	Inflammatory bowel diseases	- number of stationary treatments per year (cases) - average residence time - number of operations - inability to work - premature mortality
W-Dahl et al. 2005 [51]	Evaluation of the patient-relevant outcomes pain, function, and quality of life during two years in patients operated on for knee osteoarthritis with tibial osteotomy	Prospective follow-up study	1 term: - patient-relevant outcome	Tibial osteotomy for uni-compartmental knee osteoarthritis	- knee-specific health status: pain, symptoms, activities of daily life, sports and recreation function, knee-related quality of life [KOOS] - complications, e.g., delayed healing, deep venous thrombosis

Regarding the study design, we found a mixture of methodological approaches consisting mainly of reviews (16 of 44, 36.4%) [3–5, 18, 25, 27–29, 31–36, 40, 48] and qualitative or mixed-methods studies including Delphi approaches (12 of 44, 27.3%) [11–17, 19, 21–24]. Whereas the reviews considered studies across different countries, other studies related to a specific country were conducted mainly in Sweden (*n* = 9) [30, 37–39, 43, 45–47, 51] and Germany (*n* = 4) [11, 12, 49, 50].

### Terminology

Based on the 44 articles included in this review, we identified 35 different terms for patient-relevant outcomes (Table 5). Of these 35 terms, the two most frequently used terms were *patient-important outcome* (identified in 23 articles, 52.3%) and *patient-relevant outcome* (identified in 17 articles, 38.6%).

**Table 5:** Terms used for supposedly patient-relevant outcomes (*n* = 44 articles)

Term, identified in the (international) literature	Number of articles using this term (%)
Patient-important outcome	23 (52.3)
Patient-relevant outcome	17 (38.6)
Patient-relevant benefit	4 (9.1)
Patient preference	3 (6.8)
Patient outcome	3 (6.8)
Patient-relevant endpoint	2 (4.5)
Patient-centred outcome	2 (4.5)
Patient-reported outcome	2 (4.5)
Patient-relevant treatment benefit	2 (4.5)
Patient endpoint	1 (2.3)
Patient-prioritised outcome	1 (2.3)
Patient-preferred outcome	1 (2.3)
Patient-preferred health and treatment outcome	1 (2.3)
Patient-reported benefit	1 (2.3)
Patient-relevant health outcome	1 (2.3)
Patient-oriented outcome	1 (2.3)
Patient-relevant clinical trial outcome	1 (2.3)
Self-reported outcome	1 (2.3)
Patient-relevant measure	1 (2.3)
Self-administered outcome measure	1 (2.3)
Patient-relevant target parameter	1 (2.3)
Patient-relevant parameter	1 (2.3)
Patient-relevant efficacy parameter	1 (2.3)
Patient-important activity and participation outcome	1 (2.3)
Patient-important functional outcome	1 (2.3)
Patient-important clinical outcome	1 (2.3)
Child and family-important outcome	1 (2.3)
Patient- and caregiver-relevant outcome	1 (2.3)
Patient- and caregiver-relevant endpoint	1 (2.3)
Patient-related outcome	1 (2.3)
Patient-centered endpoint	1 (2.3)
Patients’ important outcome	1 (2.3)
Patients’ important treatment outcome	1 (2.3)
Patient priority outcome	1 (2.3)
Patient priority treatment outcome	1 (2.3)

In 21 articles (47.7%) one term was used consistently for supposedly patient-relevant outcomes; in 14 articles (31.8%) two terms were used, and in the remaining nine articles (20.5%) up to six different terms were used to describe the outcomes. We were not able to identify different patterns in terminology across countries. The terms identified per article are demonstrated in Tables 1, 2, 3 and 4.

### Outcomes

**Fig. 2:**
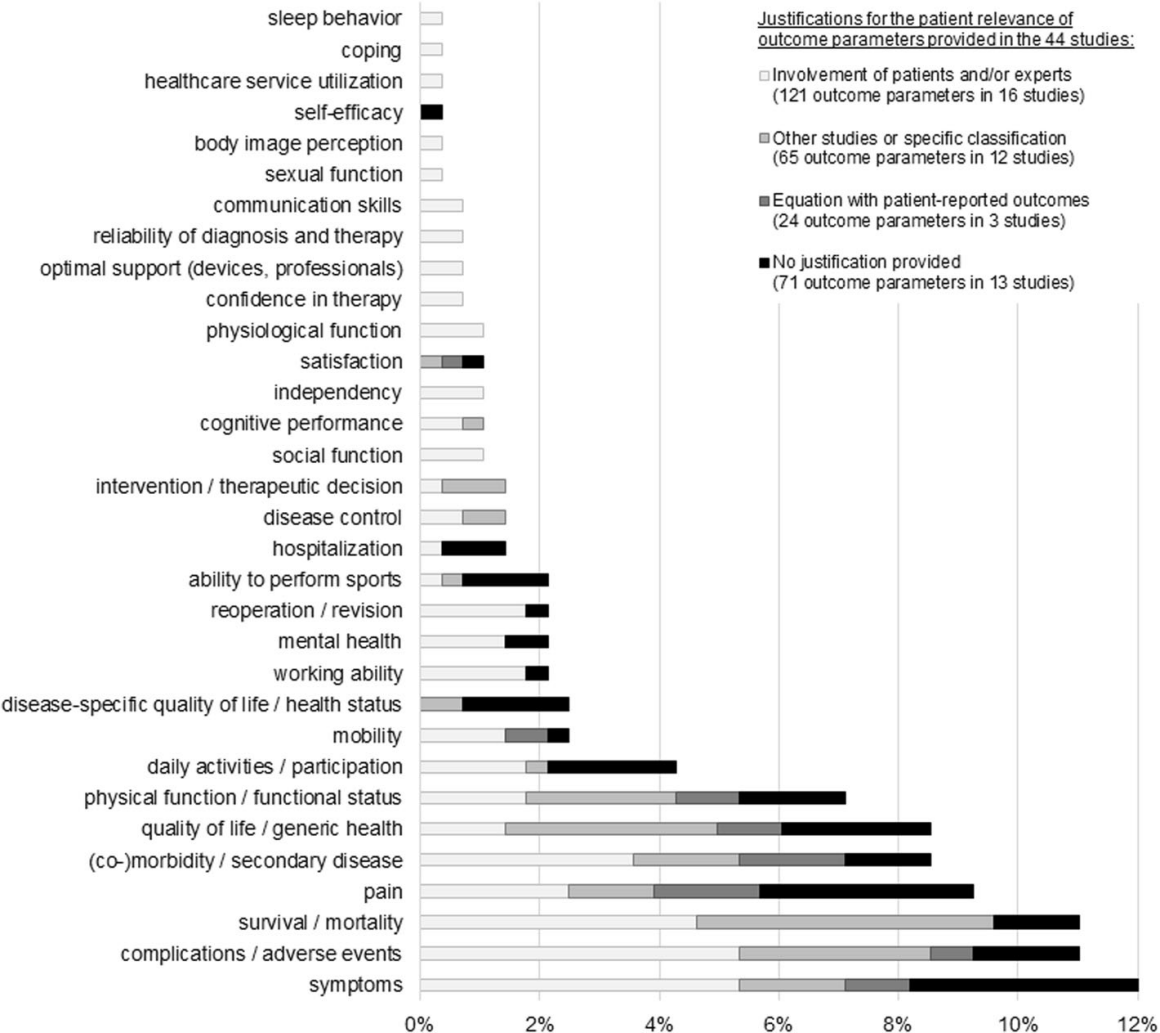
Distribution of the 281 codes representing patient-relevant outcomes in 32 inductive categories (bars) with the distribution for each category grouped by the four justifications provided for the patient relevance of outcomes (shading); the percentages on the x-axis refer to 281

A structuring content analysis of the outcomes extracted from the 44 articles resulted in 281 codes, from which 32 inductive categories were composed. Irrespective of the different shadings per bar, Fig. 2 illustrates these 32 categories in ascending order of frequency (. The most strongly represented categories were: symptoms (*n* = 34 codes, 12.1%), adverse events/complications (*n* = 31 codes, 11.0%), survival/mortality (*n* = 31 codes, 11.0%), pain (*n* = 26 codes, 9.3%), generic quality of life/health status (*n* = 24 codes, 8.5%), (co)morbidity/secondary diseases (*n* = 24 codes, 8.5%), and physical function/functional status (*n* = 20 codes, 7.1%). The outcomes extracted per article are listed in Tables 1, 2, 3 and 4.

### Justification

In about one third (*n* = 13, 29.5%) of the 44 articles analyzed, no reason was provided why the outcomes reported were considered to be relevant to patients (Table 4). However, in other studies we found different explanations justifying the selection of patient-relevant outcomes that refer mainly to three core issues:

1. Involvement of patients and/or experts was apparent in 16 out of 44 articles (36.4%) (Table 1). To explain their choice of outcomes authors referred either to patients only (*n* = 9) [13, 15–17, 20, 22–25] or to experts only (*n* = 2) [14, 26] or to both patients’ and experts’ opinion (*n* = 5) [11, 12, 18, 19, 21]. One study on early childhood disease included caregivers instead of patients [21], another one on critical disease included caregivers in addition to patients [13]. Sample sizes differed largely from smaller qualitative studies including 10 to 30 patients recruited in the health care setting [21, 22, 24] to a cross-sectional study in a random sample of about 2000 individuals invited by mail [20].
2. Referral to other studies [4, 5, 27–34] or special classification [35, 36] was used as another source of supposedly patient-relevant outcomes (Table 2). These articles (*n* = 12, 27.3%) referred their choice of outcomes mainly to reviews [4, 5, 27, 28, 34] or to a specific classification such as the International Classification of Functioning, Disability and Health [36].
3. A few articles considered patient-reported outcomes equivalent to patient-reported outcomes (*n* = 3, 6.8%; Table 3). In one of these articles authors argued that patient-relevant outcomes rely exclusively on the information provided by patients themselves [39].

The different shading of the bars in Fig. 2 illustrates the allocation of the outcomes extracted from the studies considering justifications of patient relevance provided by the authors. It shows that more popular outcome categories were justified on the basis of different explanations, whereas more seldom categories and those based on social aspects were commonly traced back to patients and/or experts.

### Subgroup analysis of articles involving patients and/or experts

All articles that had actively involved patients and/or experts focused on a specific disease (Table 1), but outcomes considered patient-relevant showed a widespread distribution with regard to the 32 inductive categories identified, referring to as many as 29 different categories. However, these studies did not include outcomes related to satisfaction, self-efficacy, or disease-specific quality of life/health status (Fig. 2). Interestingly, we found that 12 of the 32 inductive categories were only used in articles with patient and/or expert involvement such as physiological functioning, ability to fulfil social functions, independency, reliability of diagnosis and therapy, and confidence in therapy. However, outcomes most commonly described as patient-relevant, such as symptoms, adverse events/complications, survival/mortality, and (co)morbidity/secondary disease were identical to those most commonly described as patient-relevant in the other 28 articles not involving patients and/or experts (Fig. 2).

Stratifying articles involving patients and/or experts (*n* = 16) by the manner of involvement showed that survival/mortality was mentioned especially in studies involving experts only. On the other hand, outcomes identified only in studies involving patients and/or experts (i.e. physiological functioning, reliability of diagnosis and therapy, or confidence in therapy) were traced back to those studies involving either patients only or both patients and experts.

## Discussion

In this scoping review, we analyzed the international literature of the last 20 years with regard to patient relevance. We found a large variety of terms used as well as numerous supposedly patient-relevant outcomes. No more than one third of the articles referred to the patients’, the caregivers’ or the experts’ opinions to explain the relevance of the outcomes reported. All in all, we were not able to identify a consistent concept or understanding of patient-relevant outcomes. Table 6 summarizes the main findings and implications for future research.

**Table 6:** Summary of findings and implications for future research

Research question	Findings	Implications for future research
What terminology is used for supposedly patient-relevant outcomes?	- large variety of terms found in the international literature - inconsistency of terms across and within papers - possible confusion between patient-relevant and patient-reported	- consistent concept of patient relevance is needed - standard set of patient-relevant outcomes is needed - patients must be involved when defining what is relevant to them - consistency regarding patient relevance will improve the comparability of study results and enable patients to make informed choices
What outcomes are considered to be relevant to patients?	- wide range of outcomes found - commonly disease-specific outcomes - social dimensions underrepresented	
What explanations are provided to justify the relevance of these specific outcomes for patients?	- one third of studies without any explanation - another third refers to the opinion of patients, experts, or caregivers as patient representatives - outcomes exclusively defined by experts do not necessarily represent patients’ preferences	

According to our analysis, the reasons for the identified ambiguity appear to be heterogeneous. Some articles did not differentiate between patient-reported and patient-relevant outcomes [37–39]. Other articles emphasized a potential overlap of the terms, but noted that not every outcome which can be reported by patients necessarily has to be relevant for them [16, 18, 23, 24, 41]. Two articles on chronically ill children expanded the terminology from patients to the family to underline the social context and impact [21, 36]. In both studies, the target group was involved in the definition of relevant outcomes. Indeed, such patient- or target group-driven approaches are not common practice yet: two thirds of the articles we analyzed referred to previous articles [4, 5, 27–34] or did not even provide a justification as to why the reported outcomes were considered to be relevant to patients [3, 40–51]. With respect to both of these justifications, Murad et al. (2011) pointed out that neither outcomes considered patient-relevant in other studies nor those described as relevant to patients from a researchers’ perspective necessarily represent what really matters to patients [20]. In addition, our subgroup analysis on studies involving patients and/or experts suggested that outcomes defined solely by experts in sense of health care professionals might not necessarily represent the patients’ perspectives as well. Transferring this to practice implies that patient-driven approaches are required: Patients or representatives need to be asked when defining what is relevant for them. Referring to the patients’ or – in the case of young children or critical care – the caregivers’ opinions, such patient-driven approaches were identified in 14 articles included in our scoping review, but were limited to specific diseases such as cardiovascular disorders [11–13, 15–25]. For these particular patients, adverse events like myocardial infarction or stroke might be important outcomes but may not necessarily be applicable to other patient groups as specific diseases are commonly associated with very specific needs and preferences that cannot be generalized.

The number of outcomes identified in the context of patient relevance with 32 inductive categories certainly exceeded our expectations. However, outcomes representing social dimensions including the ability to perform daily activities or to fulfil social functions were apparently underrepresented, especially in studies not involving patients and experts. The variety of outcome measures as well as the lack of a consistent concept with regard to outcomes representing patient relevance limit the comparability of study results and make it difficult to determine the particular patient benefit of different interventions. This problem is also known from patient-centered care: Based on a systematic review and concept analysis of 417 articles, Scholl et al. (2014) found that models on patient-centeredness lack conceptual clarity, resulting in heterogeneous terminology and inconsistent outcome measures [52]. Even in the context of patient-relevant outcomes, this criticism was already voiced years ago, for example by Cleveringa et al. (2010) [42].

Meanwhile, promising initiatives like the European Qualitative research project on Patient-preferred outcomes in Early Rheumatoid Arthritis [53] or the International Consortium for Health Outcomes Measurement (ICHOM) [54] address this problem by defining core sets of outcomes that matter to patients. With a stronger focus on the operationalization of patient-relevant outcomes, a working group at the German Center for Health Services Research in Dermatology developed and validated a questionnaire assessing patient-relevant treatment benefits. This so-called Patient Benefit Index is available for different diseases, e.g. for the treatment of pruritus, lymphedema, and lipedema [11, 12]. While such initiatives usually focus on individual diseases, the ICHOM has also started to develop outcome sets across diseases (e.g., overall adult health, older persons, overall pediatric health) [55]. Providing validated instruments for each outcome set, the ICHOM may have the potential to standardize outcome measurement in (clinical) trials, even though the initiative originally aimed to standardize the assessment of routine care data across countries to improve the quality of healthcare for common diseases worldwide.

Based on this scoping review we aimed to improve the conceptual clarity on patient relevance. Since the results do not allow us to derive a clear concept, in a next step we plan to close this gap by conducting a study among patients, health care professionals, and researchers. Unlike the approaches described above, we not only aim to identify a core set of generic rather than disease-specific patient-relevant outcomes, but also to contribute to a clear definition and understanding thereof. Based on the findings of this review, patients and health care professionals will be involved to define outcomes that are relevant to patients across diseases and important components of an adequate definition. Researchers will additionally ensure that the concept is feasible for research purposes. Our overarching long-term objective is to increase the comparability of study results with regard to patient relevance.

### Limitations

A key strength of this scoping review is the systematic approach: The terms and outcomes were systematically and independently extracted by two researchers. Also, the literature search was not limited to fixed term sequences, but considered combinations of terms, e.g. “patient-relevant” and “outcome” instead of “patient-relevant outcome”. Due to this approach, articles with titles including a statement or question like “Are outcomes reported patient-relevant?” were also covered. Nevertheless, the search strategy focused only on the titles of articles. Thus, it cannot be excluded that we missed relevant articles. This is further aggravated by the fact that the terminology used in the literature lacks consistency. For example, we did not consider search terms like “patient-centered outcomes” as we aimed to focus on outcomes that are relevant to patients, but these terms might have been used as synonyms in some articles as well. Finally, the restriction to full-text availability might represent another limitation. However, the abstracts alone did not provide sufficient information regarding supposedly patient-relevant outcomes and outcome justification.

## Conclusions

Returning to the initial question what outcomes are particularly relevant to patients and who determines their relevance, we conclude that recent studies use a variety of outcomes without asking patients what really matters to them. We were unable to identify either a sound definition or a consistent outcome set of patient-relevant outcomes, not even in the few studies that actively involved patients. In our opinion, consensus on a consistent terminology and set of generic patient-relevant outcomes is needed to adequately operationalize patient-centered care, increase the comparability of study results, and thereby enable patients to make choices regarding therapy in the context of shared decision-making.

## Supplementary information

**Additional file 1.** Full electronic search strategy for Embase, PubMed, Cochrane Central, Scopus, and Google Scholar.

## Data Availability

All data generated or analyzed during this study are included in this published article.

## Abbreviations

DES-SF: Diabetes Empowerment Scale-Short Form
DHP-18: Diabetes Health Profile (18 questions)
DNVF: German Network for Health Services Research
DTSQ-status: Diabetes Treatment Satisfaction Questionnaire
EQ-5D: European Quality of Life questionnaire - 5 Dimensions
HADS: Hospital Anxiety and Depression Scale
IES-R: Impact of Event Scale-Revised
IQEHC: Institute for Quality and Efficiency in Health Care
KOOS: Knee Injury and Osteoarthritis Outcome Score
LOST-IT: LOST to follow-up Information in Trials
OARS questionnaire: Older American Resources and Services questionnaire
SF-36: Short Form-36 Health Survey
WOMAC: Western Ontario and McMaster Universities Osteoarthritis Index
