# Supplementary material for: Patient-relevant outcomes: what are we talking about? A scoping review to improve conceptual clarity
Source: BMC Health Serv Res. 2020 Jun 29;20:596. doi: 10.1186/s12913-020-05442-9 (PMC7325243; doi:10.1186/s12913-020-05442-9)
Supplement: Supplementary file 1 — Additional file 1. Full electronic search strategy for Embase, PubMed, Cochrane Central, Scopus, and Google Scholar. [file 12913_2020_5442_MOESM1_ESM.docx]

**Additional file 1.** Full electronic search strategy for Embase, PubMed, Cochrane Central, Scopus, and Google Scholar.

| **Database** | **Search term** | **Additional limitations** | **Last update** | **Records** |
| --- | --- | --- | --- | --- |
| Embase | ((patient-relevant or patient-important or patient-preferred) and (outcome* or endpoint* or parameter* or indicator*)).ti. | limit 1 to yr="2000 -Current" limit 2 to (english or german) | 31/07/2019 | 133 |
| PubMed | (patient-relevant [Title] OR patient-important [Title] OR patient-preferred [Title]) AND (outcome* [Title] OR endpoint* [Title] OR parameter* [Title] OR indicator* [Title]) AND ("2000/01/01"[PDAT] : "2019/07/31"[PDAT]) AND (English[lang] OR German[lang]) |  | 31/07/2019 | 76 |
| Cochrane Central | (patient-important OR patient-relevant OR patient-preferred) in Record Title AND (outcome* OR endpoint* OR indicator* OR parameter*) in Record Title | publication year from 2000 to 2019, in trials, no word variations | 31/07/2019 | 15 |
| Scopus | TITLE (patient-relevant OR patient-important OR patient-preferred) AND TITLE (outcome* OR endpoint* OR parameter* OR indicator*) PUBYEAR > 1999 AND (LIMIT-TO (LANGUAGE, "English") OR LIMIT-TO (LANGUAGE, "German")) |  | 31/07/2019 | 89 |
| Google Scholar | allintitle: (patient-relevant OR patient-important OR patient-preferred) (outcome OR outcomes OR endpoint OR endpoints OR indicator OR indicators OR parameter OR parameters) | publication year from 2000 to 2019, no citations, no patents | 31/07/2019 | 128 |
